# Nitrogen Deficiency Enhances Eggplant Defense against Western Flower Thrips via the Induction of the Jasmonate Pathway

**DOI:** 10.3390/plants13020273

**Published:** 2024-01-17

**Authors:** Yueqin Zheng, Qianxia Liu, Shuang Shi, Xiaowen Zhu, Yong Chen, Shuo Lin, Houjun Tian, Lanyan Huang, Hui Wei

**Affiliations:** 1 State Key Laboratory of Ecological Pest Control for Fujian and Taiwan Crops, Institute of Plant Protection, Fujian Academy of Agricultural Sciences, Fuzhou 350013, China; cyqzheng@163.com (Y.Z.); liuqianxia0627@163.com (Q.L.); shishuanghb@163.com (S.S.); zhuxiaowen1949@163.com (X.Z.); cheny0903@163.com (Y.C.); bushbucklinshuo@163.com (S.L.); tianhoujunbest@163.com (H.T.); huanglanyan1998@163.com (L.H.); 2 Fujian Key Laboratory for Monitoring and Integrated Management of Crop Pests, Fuzhou Scientific Observing and Experimental Station of Crop Pests of the Ministry of Agriculture, Fuzhou 350013, China; 3 College of Plant Protection, Fujian Agriculture and Forestry University, Fuzhou 350002, China

**Keywords:** induced defense, JA, N deficiency, eggplant, anti-herbivore resistance, western flower thrips

## Abstract

Plant nutrition is connected to defense against insect herbivores, but the exact mechanism underlying the effect of the nitrogen (N) supply on the anti-herbivore capacity of eggplants (*Solanum melongena*) has not been studied in detail. Therefore, we examined the impact of low (LN, 0.5 mM) and high (HN, 5 mM) nitrate levels on eggplant resistance against the western flower thrips *Frankliniella occidentalis* (WFT), a major destructive eggplant pest. Our results showed that LN plants displayed enhanced defense responses to WFT compared to HN plants. This included increased transcript levels of key genes in the jasmonic acid (JA) pathway, the accumulation of JA-amido conjugates (jasmonoyl-isoleucine, jasmonoyl-phenylalanine, and jasmonoyl-valine), JA precursor (12-oxophytodienoic acid), and methyl jasmonate, higher transcript levels of defense marker genes (*MPK3*, *MPK7*, and *WRKY53*), and increased activities of polyphenol oxidase and peroxidase upon a WFT attack. Our findings suggest that N deficiency can prime JA-mediated defense responses in eggplants, resulting in increased anti-herbivore resistance.

## 1. Introduction

Many rice-cultivating regions of Asia have experienced increases in the abundance of major insect pests due to the extensive application of N fertilizers [1]. While N fertilizers enhance crop productivity, their excessive application can have detrimental effects on the environment. N is an essential macronutrient [2] and is the most common limiting factor for the growth and development of both plants and herbivores. Furthermore, N can act as a signaling molecule, influencing gene expression and various aspects of plant metabolism, physiology, growth, and development [3,4], particularly when plants are subjected to biotic stress [5,6,7]. High N levels in plants can lead to a faster weight gain and shorter life cycles in insect herbivores, resulting in increased survival, reproduction, and population sizes [8,9,10]. Conversely, low N levels in plants can lead to the stunted growth of insect herbivores, who experience a decreased pupal weight and an extended developmental period from the egg stage to the adult stage [11]. Plants grown in low-N media undergo metabolic remodeling, including an increase in the accumulation of constitutive phenolics and tomatine, which potentially confers greater resistance to herbivores [5,11,12]. Although numerous studies have shown that plants with low levels of N are more resistant to insect damage, the exact mechanism underlying this interaction between plants and insects at varying N levels remains unclear.

Plants have developed a range of strategies, including constitutive and induced defenses, to protect themselves from phytophagous insects [13]. Trade-offs in these two types of defense exist among species, which can be beneficial for plants [14,15]. Constitutive defense is expressed by default, whereas induced defense is activated only upon herbivore attack [16]. The jasmonic acid (JA) signaling pathway serves as a regulatory system which plants use to defend themselves from chewing insects and consume cell contents feeders such as thrips [17,18,19,20]. In contrast, the salicylic acid (SA) signaling pathway is assumed to regulate induced defense responses against phloem feeders [21,22]. According to Thaler et al. [23] and Caarls et al. [24], JA and SA may interact either antagonistically, synergistically, or additively depending on the plant species. Upon an attack by insect herbivores, plants can recognize certain molecular patterns that are specific to herbivores, activate a mitogen-activated protein kinase (MAPK) cascade, initiate JA/SA signaling, and increase the expression of defense-related genes and the biosynthesis of defense-related metabolites [25]. Having an inducible defense system is beneficial in that it enables plants to be more economical with their energy resources by synthesizing defense compounds only when needed [16,26]. In addition, plants have developed a defense-priming strategy that is initially provoked by chemical and biological stimuli or other stresses, allowing plants to react rapidly and effectively to potential threats [27]. For instance, N deficiency can bolster the defense of rice against striped stem borer *Chilo suppressalis* infestation by increasing the levels of phenolic acids, flavonoids, and JA [7]. Moreover, Si can prime JA-mediated rice defense against the rice leaffolder *Cnaphalocrocis medinalis* [26]. Currently, it remains uncertain how N deficiency can prime eggplant’s defense against thirps infestation.

Eggplant (*Solanum melongena* L.), a crop in the family Solanaceae with great economic importance, is consumed and grown worldwide [28,29]. Its yield is susceptible to N fertilization and can be severely damaged by thrips [30,31]. The western flower thrips *Frankliniella occidentalis* (Pergande) (Thysanoptera: Thripidae) (WFT) is a destructive insect pest that can cause great economic losses owing to its polyphagous nature and ability to pierce plants and suck out their cell contents [19,32,33]. This insect is also a vector for the tomato spotted wilt virus (TSWV) [34,35]. The feeding of thrips present on sweet pepper (*Capsicum annuum* L.) can lead to an increase in the expression of JA-related genes such as *CaLOX2* and *CaPIN II* [35]. The external application of JA/MeJA is effective at impairing WFT feeding [36] and prolonging their growth phase [37].

In this study, we investigated the relationship between nitrate supply and eggplant’s defense against WFT through JA-mediated anti-herbivore mechanisms. We examined the damage caused by thrips and their preference for eggplants that were treated with different N concentrations. Additionally, we analyzed the changes in hormone pathways using transcriptome analysis after thrips feeding. Specifically, we focused on the introduction of JA- and SA-regulated genes, as well as JA- and SA-related hormones, in response to thrips feeding. Through our research, we identified key genes, marker genes, and defense enzyme activity in plants exposed to low nitrate (LN) and high nitrate (HN) with either exogenous MeJA or thrips feeding. This study aimed to explore the role of JA signaling in the defense response of eggplants to thrips feeding under short-term N treatment.

## 2. Materials and Methods

### 2.1. Plant Cultivation

Eggplant seeds of the cultivar *Solanum melongena* L. cv. Hangqie 1 were surface sterilized with 5% (*v*/*v*) NaClO for 20 min and rinsed three times with conductivity water. 

After being sterilized, the seeds were soaked in conductivity water for two days in darkness, after which they were placed in a seed tray in a constant temperature incubator for 10 d. Following germination, the seedlings were shifted to a black hydroponic tank and cultured for 15 d with 2.5 mM K_2_SO_4_, 0.5 mM Ca(NO_3_)_2_, 1 mM KH_2_PO_4_, 2 mM MgSO_4_, 0.32 μM CuSO_4_, 0.76 μM ZnSO_4_, 20 μM Fe(II)-EDTA, 46 μM H_3_BO_3_, 0.11 μM Na_2_MoO_4_, and 9 μM MnCl_2_ [29]. The tanks were stored in a greenhouse with a temperature regime of 24 °C for 14 h and 22 °C for 10 h, and the light intensity was 35,000 lux. The nutrient solution was changed every 3–4 d, and the pH was kept at 5.8 through the daily addition of 1 N KOH. Then, the seedlings were moved to a nutrient solution with either 0.25 mM Ca(NO_3_)_2_ or 2.5 mM Ca(NO_3_)_2_ and cultured for a further 5 d. For the LN treatment, 2.25 mM calcium sulfate was used to replenish calcium levels [38].

### 2.2. WFT Treatment

The WFT were provided by the Institute of Plant Protection, Hunan Academy of Agricultural Sciences, China. Species identification was confirmed through quantitative real-time PCR (qRT-PCR). The thrips were fed broad beans (*Vicia faba* L.) under the conditions of 25 °C, a 14:10 (L:D) h photoperiod, 65 ± 5% RH, and a light intensity of 4800 lux [39]. After 5 d of exposure to various concentrations of nitrate, the plants were inoculated with female adult WFT in both selective and non-selective experiments to observe infestation. One hundred female adult WFT were introduced to the plants, and the selection experiment involved observing and recording the number of thrips on each plant after 3 d. The petiole of the third true leaf of an eggplant seedling was inserted into a container containing 1% agar solution and inoculated with five female WFT onto the leaf for a non-selective experiment. After 24 h, the size of the feeding scars on the leaves was determined [35].

### 2.3. RNA Sequencing

Plants were grown for 20 d and then exposed to female adult WFT for 24 h, followed by the extraction of total RNA using TRIzol reagent (Invitrogen, Santa Clara, CA, USA). The required fragments were sequenced using the Illumina Novaseq TM 6000 platform (OE Biotech, Inc., Shanghai, China). The reference genome was acquired as previously described [40]. The Fastp Toolkit was used for the quality control of raw reads, which were then aligned to the reference genome [41]. The R package DESeq2 was used to detect the differentially expressed genes (DEGs) [42]. All the unigenes were further annotated using GO and KEGG pathway analyses.

### 2.4. Quantitative Real-Time PCR Analysis

To verify the differential expression of the selected genes, qRT-PCR was carried out [7]. The total RNA was obtained from the treated leaves by employing Eastep^®^ Super Total RNA Extraction Kit (Progema, Shanghai, China). The HiScript^®^ II 1st Strand cDNA Synthesis Kit (Vazyme Co., Ltd., Nanjing, China) was then employed to generate cDNA from 1 µg of RNA, and qPCR was performed using the Taq Pro Universal SYBR qPCR Master Mix (Vazyme Co., Ltd., Nanjing, China). To assess the qPCR amplification, an internal control was used in the form of *SmActin* (GenBank accession number: GU984779.1). The primer sequences used are mentioned in Supplementary Tables S1 and S2. Three biological replicates, each with five plants from each treatment, were evaluated through qRT-PCR, and changes in the expression of the gene were determined using the 2^−ΔΔCt^ method with normalization [35].

### 2.5. Plant Hormone Analysis 

Eggplant seedlings were cultivated under different nitrate concentrations for 5 d. Leaf tissues were collected 0, 3, 9, and 24 h following WFT infestation. The leaves were then frozen in liquid nitrogen and kept at a low temperature of −80 °C for preservation. Five plants were selected, and three replicates were established for each sample over each period. A 50 mg plant sample was blended with a 15:4:1 (*v*/*v*) ratio of methanol, water, and formic acid. The extract was combined with 10 μL of a 100 ng/mL internal standard solution and then mixed for 10 min before being centrifuged for 5 min at 12,000 r/min and 4 °C. The supernatant was transferred to a plastic microtube, evaporated, and mixed with 80% methanol (*v*/*v*) to 100 μL. A 0.22 μm membrane filter was utilized to filter the solution, and the LC-MS/MS analysis was used to measure the concentrations of JA, dihydro jasmonic acid (H_2_JA), jasmonoyl-isoleucine (JA-Ile), jasmonoyl-phenylalanine (JA-Phe), jasmonoyl-valine (JA-Val), 12-oxophytodienoic acid (OPDA), methyl jasmonate (MeJA), SA, and salicylic acid 2-o-β-glucoside (SAG) [43,44].

### 2.6. MeJA Treatment

In this experiment, eggplants were treated with MeJA, which was converted to JA and then to its bioactive form JA-Ile [18]. MeJA (Coolaber, 98%) was dissolved in 0.2% ethanol and then further diluted to a concentration of 100 μM in double-distilled water [45]. The solution of 100 μM MeJA or an equal volume of ethanol was sprayed onto LN/HN-treated eggplants. Following treatment, the third true leaves were collected at 0, 1.5, 3, 6, 9, and 24 h, frozen in liquid nitrogen, and kept at −80 °C. Five plants were selected from each sample, and three replicates were established for every time period.

### 2.7. Determination of Polyphenol Oxidase and Peroxidase Activities

Samples of leaves were taken 0, 9, and 24 h after either MeJA treatment or WFT infestation and used to analyze polyphenol oxidase (PPO, EC1.10.3.1) and peroxidase (POD, EC 1.11.1.7) activities. Fresh samples, approximately 1 g in weight, were frozen and then ground until they became a fine powder. This powder was homogenized with a 0.05 M phosphate buffer (pH 7.8; PPO, pH 7.2 POD) that had 1% (*w*/*v*) polyvinylpyrrolidone. The mixture was then centrifuged at 4 °C for 15 min at 12,000× *g*, and the supernatant was utilized for enzyme assays [7]. PPO activity was assayed as described by Zauberman et al. [46], and POD activity was assayed as described by Kraus and Fletcher [47]. For each treatment, three independent biological replicates were conducted, and the absorbance change at 525/470 nm was determined to be 0.01/0.005, which was used as the unit of enzyme activity per minute per gram of tissue per milliliter of the reaction system.

### 2.8. Data Analysis

IBM SPSS Statistics version 21 (SPSS, Chicago, IL, USA) was used for statistical analysis. Before the statistical analysis, gene transcript and hormone levels data were log-transformed. The preference data of thrips were expressed as the proportion of thrips in either treatment, with each plant being a biological replicate. To compare the means of each treatment, Student’s *t*-tests were applied.

## 3. Results

### 3.1. Low Nitrate Augments Eggplant Anti-Herbivore Defense

**Figure 1:**
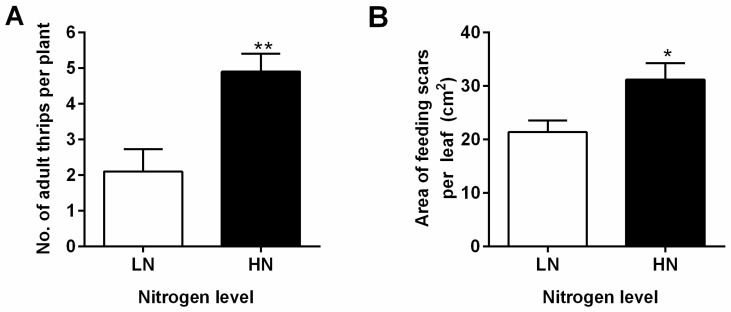
Choice (preference) (**A**) and no-choice (thrips feeding damage) (**B**) tests of western flower thrips (WFT) on eggplants cultivated under distinct concentrations (0.5 and 5 mM) of nitrate. Fifteen-day-old seedlings were transplanted to a nutrient solution containing 0.25 mM Ca(NO_3_)_2_ (LN) or 2.5 mM Ca(NO_3_)_2_ (HN) and cultured for 5 d. In the LN treatment, calcium sulfate was used to replenish calcium levels. Female adults were used for bioassays. The number of thrips adults and the feeding damage were measured 3 d (**A**) and 24 h (**B**) after inoculation. The mean ± SE was based on 10 and 15 biological duplications for the thrips population and feeding damage, respectively. For significant differences from the indicated samples: * p < 0.05; ** p < 0.01 from Student’s *t*-test.

To analyze the effects of N levels on eggplant defense against herbivores, 15-day-old eggplant seedlings were transplanted into nutrient solutions with either 0.25 mM Ca(NO_3_)_2_ (LN) or 2.5 mM Ca(NO_3_)_2_ (HN) and grown for 5 d; then, they were infested with female WFT. The results showed that the LN treatment decreased the seedling dry weight, leaf length, and leaf width, whereas there was no significant difference in shoot or root length compared to those in the HN treatment (Figure S1). WFT adults showed a preference for HN-cultured plants 3 d after inoculation (Figure 1A). WFT feeding on the leaves of plants cultured under LN and HN conditions damaged 21.4 cm^2^ and 31.2 cm^2^ of the leaves, respectively (Figure 1B). This implies that LN application can enhance eggplant defense against WFT infestation.

### 3.2. Effect of Nitrate Levels on the Eggplant Phytohormone Response to WFT Infestation

**Figure 2:**
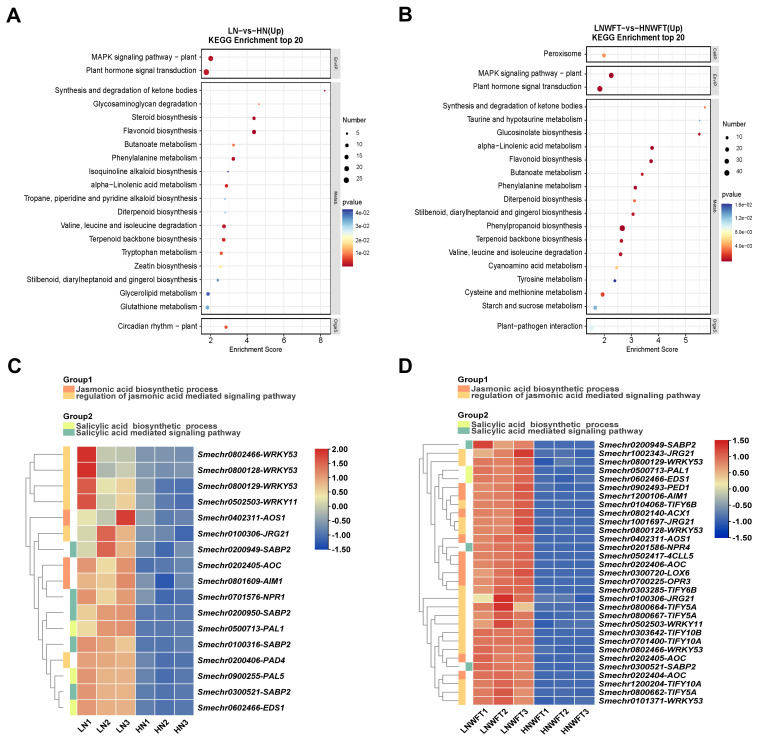
Transcriptome analysis of the leaves of eggplants cultivated with different concentrations (0.5 and 5 mM) of nitrate and inoculated with WFT. Fifteen-day-old seedlings were moved to a nutrient solution with either 0.25 mM Ca(NO_3_)_2_ (LN) or 2.5 mM Ca(NO_3_)_2_ (HN) and cultivated for 5 d and then inoculated with WFT for 24 h for transcriptome analysis. KEGG enrichment analysis of the top 20 upregulated genes between the LN and HN treatments (**A**) and LNWFT and HNWFT treatments (**B**). Heatmap analysis of the jasmonic acid (JA) biosynthetic process, the regulation of the JA-mediated signaling pathway, the salicylic acid (SA) biosynthetic process, and the SA-mediated signaling pathway of upregulated genes between LN and HN treatments (**C**) or LNWFT and HNWFT treatments (**D**). For comparison, the gene expression levels were standardized. LN, 0.25 mM Ca(NO_3_)_2_ for 5 d; HN, 2.5 mM Ca(NO_3_)_2_ cultured for 5 d; LNWFT, 0.25 mM Ca(NO_3_)_2_ cultured for 5 d and then infested with WFT for 24 h; HNWFT, 2.5 mM Ca(NO_3_)_2_ cultured for 5 d and then infested with WFT for 24 h.

RNA-Seq data were analyzed to investigate the effect of nitrate supply on eggplant gene expression under WFT infestation. Quality control metrics were visualized using PCA, with correlations between the various treatments displayed in heat maps (Figure S2). The results identified 3711 and 4573 DEGs (q value < 0.05; fold change > 2) between the LN and HN treatments and LNWFT and HNWFT treatments, respectively (Figure S2C). Additionally, 674 DEGs were co-expressed between the different treatments (Figure S2D), and the results of RNA-Seq were consistent with those of qPCR (Table S1). The expression of genes related to plant hormone signal transduction was significantly higher in LN and LNWFT than in HN and HNWFT. The MAPK signaling pathway and phenylpropanoid biosynthesis were also enriched (Figure 2A,B). Genes associated with JA and SA biosynthesis and signaling pathways were also upregulated in LN and LNWFT compared to those in HN and HNWFT (Figure 2C,D).

**Figure 3:**
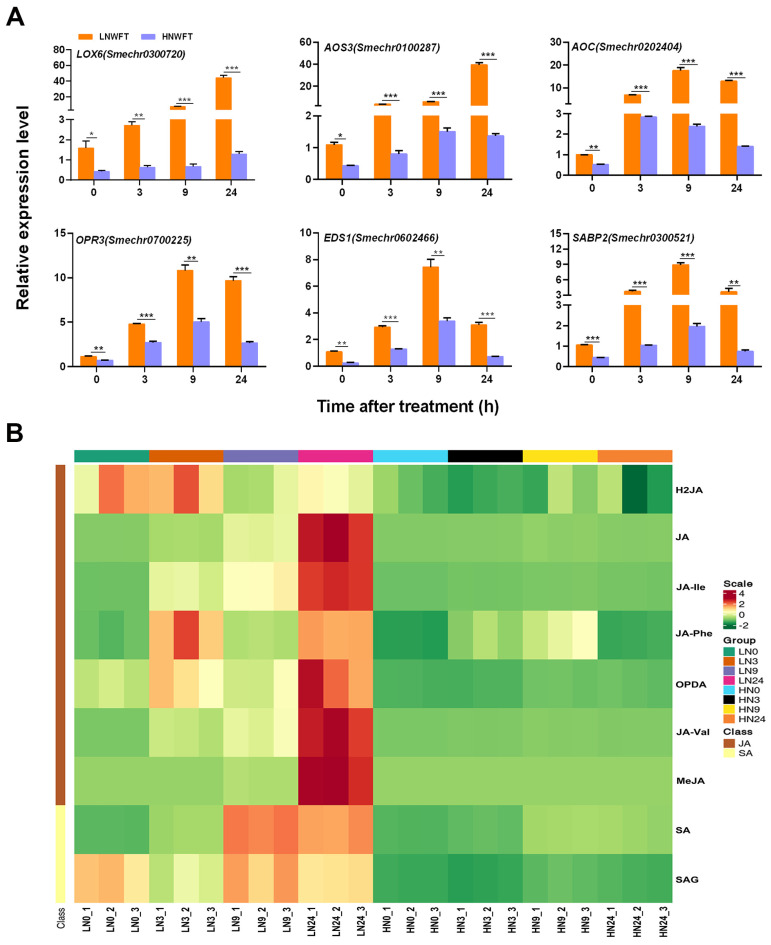
Relative expression levels of phytohormone biosynthetic and phytohormone-mediated signaling pathway genes and phytohormone levels in the leaves of eggplants cultivated with different concentrations (0.5 and 5 mM) of nitrate and inoculated with WFT. (**A**) Gene expression levels of *LOX6*, *AOS3*, *AOC*, *OPR3*, *EDS1*, and *SABP2* at 0, 3, 9, and 24 h in WFT-inoculated eggplant leaves. (**B**) Phytohormone content of JA, H_2_JA, JA-Ile, JA-Phe, JA-Val, OPDA, MeJA, SA, and SAG measured through LC-MS/MS at 0, 3, 9, and 24 h after WFT inoculation under N supply. Values are the mean ± SE (n = 3). For a significant difference from the indicated samples: * p < 0.05, ** p < 0.01, or *** p < 0.001 by Student’s *t*-test. LNWFT, 0.25 mM Ca(NO_3_)_2_ for 5 d, infested with WFT; HNWFT, 2.5 mM Ca(NO_3_)_2_ for 5 d and then infested with WFT.

To validate our findings, qPCR analysis was performed on selected genes involved in JA and SA synthesis and signaling pathways using *Smactin* as an endogenous control (Table S2). Compared with the HN treatment, the LN treatment had a considerable effect on the steady-state transcript levels of *LOX6*, *AOS3*, *AOC*, *OPR3*, *EDS1*, and *SABP2* in leaves at 0, 3, 9, and 24 h, with and without WFT (Figure 3A). Using LC-MS, the effects of 0.5 mM and 5 mM of nitrate on phytohormones were evaluated in eggplants inoculated with WFT. Phytohormone standard curves, linear equations, and correlation coefficients are shown in Table S3. Similarly, the levels of JA, JA-Ile, JA-Phe, JA-Val, OPDA, MeJA, SA, and SAG were higher in LN-cultured plants following WFT inoculation than in HN-cultured plants. JA, JA-Ile, JA-Val, OPDA, and MeJA peaked 24 h following pest infestation in the LN treatment, whereas SA and SAG peaked at 9 h. Additionally, the LN treatment significantly changed the concentrations of JA, H_2_JA, JA-Ile, JA-Phe, OPDA, SA, and SAG compared with those in the HN treatment (Figure 3B, Table S4). We suggest that JA and SA signaling are critical for eggplant defense against WFT under LN conditions.

### 3.3. Effect of Nitrate Supply on JA Biosynthesis Pathway Genes under Exogenous MeJA Application

**Figure 4:**
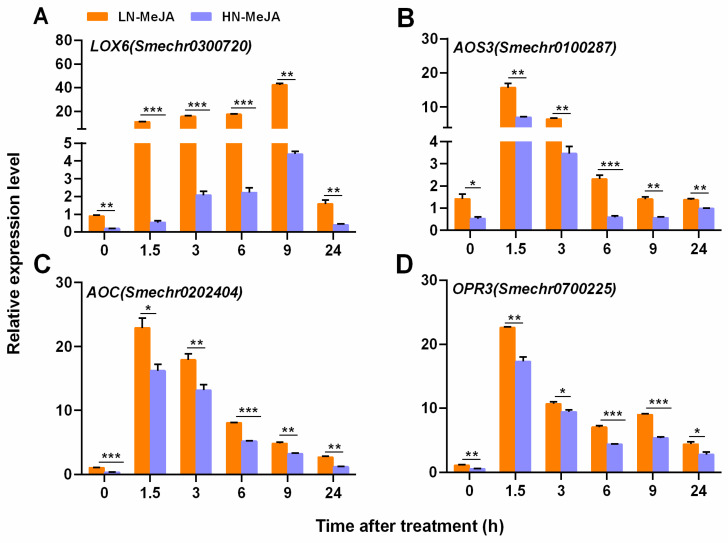
Steady-state levels of *LOX6* (**A**), *AOS3* (**B**), *AOC* (**C**), and *OPR3* (**D**) transcripts in the leaves of eggplants cultivated with two different concentrations (0.5 and 5 mM) of nitrate and sprayed with MeJA. Fifteen-day-old seedlings were moved to a nutrient solution with either 0.25 mM Ca(NO_3_)_2_ (LN) or 2.5 mM Ca(NO_3_)_2_ (HN) and cultivated for 5 d and then treated with 100 μmol MeJA. The data are displayed as the mean ± SE, based on three replicates. For significant differences from the indicated samples: * p < 0.05, ** p < 0.01, or *** p < 0.001 from Student’s *t*-test. LN-MeJA, 0.25 mM Ca(NO_3_)_2_ cultured for 5 d and then treated with MeJA for 0, 1.5, 3, 6, 9, and 24 h; HN-MeJA, 2.5 mM Ca(NO_3_)_2_ cultured for 5 d and then treated with MeJA for 0, 1.5, 3, 6, 9, and 24 h.

JA is a major component of the protective system against herbivores in eggplants. In order to further investigate the interaction between nitrate and JA, we monitored the endogenous levels of *LOX6*, *AOS3*, *AOC*, and *OPR3* transcripts in plants exposed to exogenous MeJA, with LN- and HN-treated plants as the subjects. The results showed that MeJA application resulted in an increase in the levels of all four transcripts in both types of plants, but the response was more pronounced in LN-treated plants. For instance, 1.5 h after MeJA application, the transcript levels of *AOS3*, *AOC*, and *OPR3* in LN plants were 2.2-, 1.4-, and 1.3-fold higher than those in HN plants, respectively. Furthermore, the transcript level of *LOX6* in LN plants was 9.6-fold higher than that in HN plants after MeJA application for up to 9 h (Figure 4). 

### 3.4. Low Nitrate-Mediated Enhancement of MAPK and WRKY Transcriptional Responses

**Figure 5:**
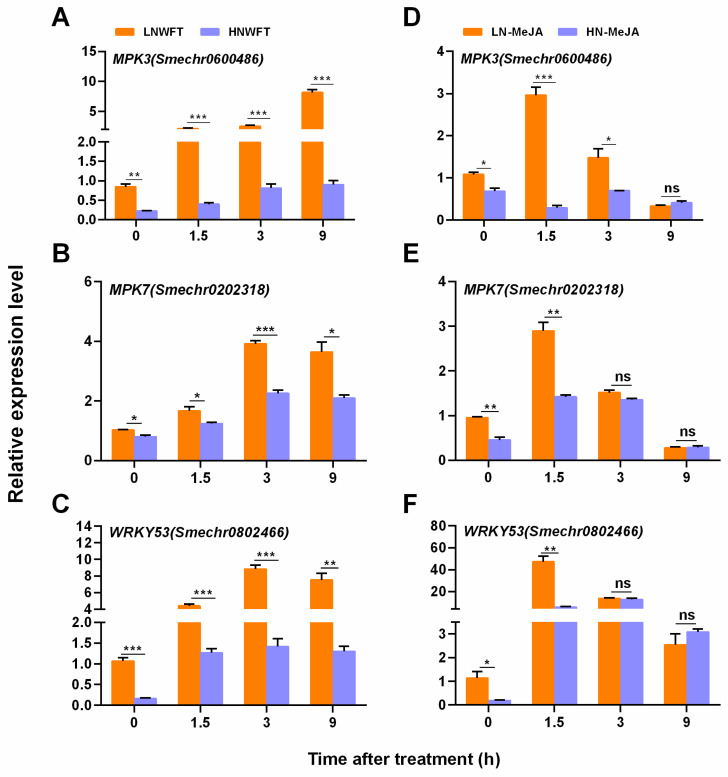
Relative expression levels of *MPK3* (**A**,**D**), *MPK7* (**B**,**E**), and *WRKY53* (**C**,**F**) genes in the leaves of eggplants cultivated with different concentrations (0.5 and 5 mM) of nitrate plus WFT or MeJA. Fifteen-day-old seedlings were moved to a nutrient solution with either 0.25 mM Ca(NO_3_)_2_ (LN) or 2.5 mM Ca(NO_3_)_2_ (HN) and cultivated for 5 d and then treated with 100 μmol MeJA or infested with WFT for 0, 1.5, 3, and 9 h. The data are displayed as the mean ± SE, based on three replicates. For significant differences from the indicated samples: * p < 0.05, ** p < 0.01, or *** p < 0.001 from Student’s *t*-test; ns indicates no significant difference. LNWFT, 0.25 mM Ca(NO_3_)_2_ for 5 d, infested with WFT; HNWFT, 2.5 mM Ca(NO_3_)_2_ for 5 d and then infested with WFT. LN-MeJA, 0.25 mM Ca(NO_3_)_2_ for 5 d and then treated with MeJA; HN-MeJA, 2.5 mM Ca(NO_3_)_2_ for 5 d and then treated with MeJA.

MAPK cascades and WRKY transcription factors are essential for plant signal transduction, especially those involved in defense against herbivores [18,48]. The steady-state levels of transcripts encoded by *MPK3*, *MPK7*, and *WRKY53* were monitored in eggplant leaves exposed to WFT infestation and MeJA treatment under LN and HN regimes (Figure 5). LN plants exposed to WFT infestation and MeJA application showed higher transcript levels of all three tested genes than HN plants. The transcript levels of *MPK3*, *MPK7*, and *WRKY53* in LN plants increased by 3.1-, 1.7-, and 6.2-fold, respectively, 3 h after WFT infestation, compared to those in HN plants (Figure 5A–C). Furthermore, a faster response to MeJA exposure compared to WFT attack was observed in LN-cultivated plants. The highest levels of *MPK3*, *MPK7*, and *WRKY53* transcripts were observed 1.5 h after MeJA exposure (Figure 5D–F).

### 3.5. Low Nitrate-Mediated Enhancement of Defense-Related Enzyme Induction

**Figure 6:**
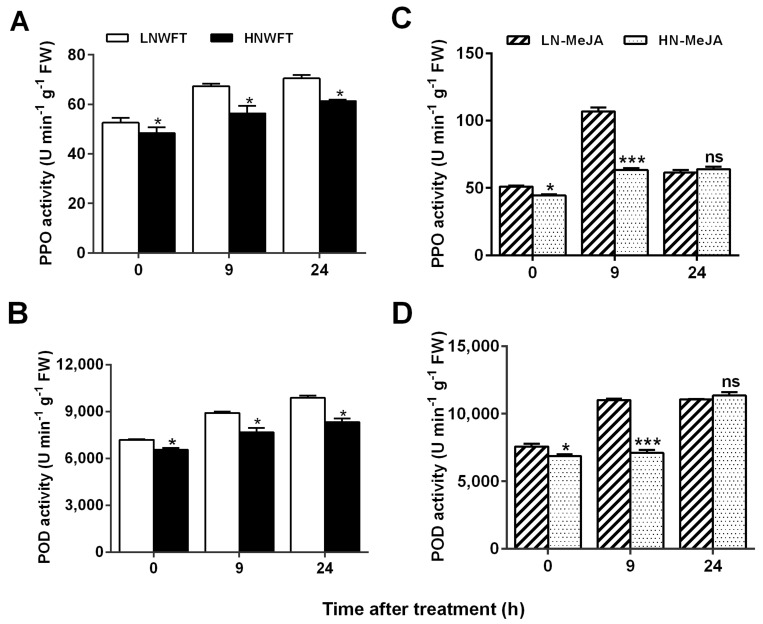
Activity levels of polyphenol oxidase (PPO) (**A**,**C**) and peroxidase (POD) (**B**,**D**) in leaves of eggplants cultivated with different concentrations (0.5 and 5 mM) of nitrate plus WFT or MeJA. Fifteen-day-old seedlings were moved to a nutrient solution with either 0.25 mM Ca(NO_3_)_2_ (LN) or 2.5 mM Ca(NO_3_)_2_ (HN) and cultivated for 5 d and then treated with 100 μmol MeJA or infested with WFT for 0, 9, and 24 h. The data are displayed as the mean ± SE, based on three replicates. For significant difference from the indicated samples: * p < 0.05, or *** p < 0.001 from Student’s *t*-test; ns indicates no significant difference. LNWFT, 0.25 mM Ca(NO_3_)_2_ for 5 d, infested with WFT; HNWFT, 2.5 mM Ca(NO_3_)_2_ for 5 d and then infested with WFT. LN-MeJA, 0.25 mM Ca(NO_3_)_2_ for 5 d and then treated with MeJA; HN-MeJA, 2.5 mM Ca(NO_3_)_2_ for 5 d and then treated with MeJA.

PPO and POD are important components of plant defense systems. Herbivore infestations can lead to increased levels of PPO and POD in plants [7,18]. To further explore the effects of N deficiency on eggplant defense responses associated with JA signaling, LN- and HN-cultivated plants were subjected to either WFT attack or MeJA treatment. Subsequently, the activities of PPO and POD were analyzed in the leaves of each experimental group (Figure 6A–D). Importantly, LN treatment increased the activity levels of PPO and POD in eggplant seedlings compared to HN treatment at both 9 and 24 h after WFT inoculation. Prior to WFT infestation, LN-cultivated seedlings showed higher levels of PPO and POD than HN-treated seedlings, which was in line with the JA levels (Figure 3B). Despite there being no significant difference in PPO and POD activity after 24 h of MeJA treatment, LN-treated seedlings displayed 68.7% and 55.2% increases in PPO and POD activity, respectively, compared with HN-treated seedlings 9 h after MeJA exposure. Overall, the LN-cultivated seedlings showed an enhanced response to both WFT attack and MeJA treatment, with MeJA treatment appearing to be a faster inducer of these responses than WFT attack.

## 4. Discussion

Plant hormones play crucial roles in plant growth, development, and responses to environmental stress. Nutrient signals can modify plant hormones [49]. Nitrate has effects on several signaling hormones including auxin [50,51], cytokinin [52,53], ethylene [54], abscisic acid [55,56], gibberellin [57], SA [58], and JA [6,59]. The NPF family of *Arabidopsis* has been identified to transport ABA, GA, and JA-Ile [60], and it can also act as auxiliary transporters when nitrate levels are low [61]. Wu et al. [62] found that JA-regulated plastid degradation and the upregulation of GDH2 dehydrogenase contribute to the release of N in rice leaves and promote the production of protective compounds/elements in rice plants under N-scarce conditions. Our previous research showed that JA and SA levels were higher in plants supplied with low N levels than in those supplied with high N levels prior to infestation with striped stem borers and that low N can prime against infestation with this insect [7]. Pi deficiency activates the JA pathway [63,64] and increases resistance to insects and pathogens in *Arabidopsis* [65,66]. Similarly, Si supplementation has been observed to induce the priming of JA-mediated defensive responses when the plants are attacked by phytophagous insects [26,67]. Additionally, low levels of K were found to reduce the damage caused by thrips compared with higher levels of K [68], demonstrating the role of JA in increasing the defensive capabilities of plants that are deficient in K. In the present study, a 5 d period of LN treatment was found to enhance the resistance of eggplants to WFT. N deprivation decreased the number of WFT adults and the associated damage (Figure 1) and increased the levels of JA and SA and the expression of these pathway genes before and after WFT infestation (Figure 2 and Figure 3, Table S4). Furthermore, the exogenous application of MeJA and WFT increased the expression of JA pathway genes and enhanced defense-related enzymes, while low N levels appeared to intensify JA-induced defense responses, acting as a JA activator (Figure 4, Figure 5 and Figure 6).

Nutrient availability can regulate plant trade-offs between growth and defense. When excessive amounts of N fertilizer are applied, the capabilities of insects can be enhanced, thus increasing their chances of survival, growth, reproduction, and population size [8,69]. A study on cranberry plants found that increased N availability reduced the resistance of plants to three types of herbivores, regardless of the genotype [70]. Another study observed that barley leaves with insufficient N had high concentrations of amino acids, sugars, and tricarboxylic acid cycle byproducts. However, when aphid larvae were transferred to these leaves, their development was hindered, and they were unable to reach full maturity [5]. Huang et al. [71] reported that exogenous calcium suppresses oviposition choices in WFT. Our study also revealed that N deprivation reduced the number of WFT adults and the associated damage (Figure 1), which is in accordance with the results of many other studies.

Our findings clearly showed that the expression of JA- (*LOX6*, *AOS3*, *AOC*, and *OPR3*) and SA- (*EDS1*, and *SABP2*) related genes was significantly higher in LN plants than in HN plants at all times following thrips feeding (Figure 3). Similar expression patterns have been observed in *Arabidopsis* [17,72], sweet pepper [35], and other plants, suggesting a crosstalk between SA and JA [23,73,74]. For example, SA can delay jasmonate-induced leaf senescence [75], and SA-induced *Arabidopsis* glutaredoxin can interact with TGA factors to inhibit JA-responsive PDF1.2 translation [76]. Our study also revealed that SA and JA levels were increased following WFT feeding, with the highest levels observed at 9 h and 24 h, respectively (Figure 3B). Abe et al. [72] observed that SA-related gene expression was higher in thrips carrying the TSWV than that of JA-related genes after feeding. Additionally, TSWV infection alone significantly increased the endogenous levels of both SA and JA, with the increase in SA being significantly higher than that of JA. Interestingly, when thrips fed on plants infected with TSWV, the JA level in the plants increased more than that observed when plants were infected with TSWV but not fed on by thrips. In contrast, the SA levels in plants infected with TSWV were comparable, regardless of the occurrence of thrips. Thrips could be manipulating plant defenses through the induction of SA, which stimulates antagonistic crosstalk with the JA pathway to disrupt plant defense responses [35,72,77]. 

This study strongly indicates the role of N deficiency in priming JA-mediated inducible defense responses, in addition to being a component of the constitutive defense that restricts feeding [5,6,7]. An analysis of JA-dependent defense-associated enzymes and genes showed that N deficiency consistently increased the induction response to insect herbivory and exogenous MeJA (Figure 4, Figure 5 and Figure 6). Beckers et al. [78] established a link between priming and MAPKs, which are essential for cellular signal amplification and can prime plants to strengthen their defense and immunity [79,80]. This activates a battery of defense genes that are often targets of WRKY-type transcription factors [81]. Our findings indicate that LN conditions induce the expression of *MPK3*, *MPK7*, and *WRKY53* when plants are subjected to WTF infestation or exogenous MeJA application. The LN plants displayed a higher transcriptional response and faster induction than the HN plants (Figure 5). Notably, the response times of JA-dependent defense-associated enzymes and genes differed when exposed to MeJA alone or fed with thrips. For instance, *MAPK* and *WRKY* genes displayed peak reactions when fed to thrips for 3 h and 9 h, whereas the highest response to MeJA alone was observed at 1.5 h, followed by a decline. Additionally, PPO and POD enzymes were elevated when fed to thrips for up to 24 h, whereas MeJA alone was effective for up to 9 h (Figure 6). A previous study had similar results, finding that the localized infection of *Arabidopsis* by *Pseudomonas syringae* pv. tomato DC3000 containing *avrRpt2* led to ongoing SA build-up throughout the plant, presumably through the sustained triggering of *MPK3/6* genes, the continual storage of dormant MPK3/6 proteins, and extended defense conditioning [80]. In conclusion, MeJA treatment alone induced defense earlier than thrips infestation alone. 

Our research revealed that N deficiency can enhance or prime JA-induced responses to herbivory, including an increased induction of JA-related genes and defense-related enzymes, as well as increased JA accumulation following an insect attack (Figure 3, Figure 4, Figure 5 and Figure 6). Insect herbivory has a major effect on the ecology and evolution of plant populations [82]. N is a major factor in the development of both herbivores and plants [1,8,69]. Our research revealed that a lack of N can affect the growth and defense of plants by activating JA-mediated anti-herbivore defense responses, which could have broad ecological and evolutionary effects on plant populations [65].

Although several studies have used JA mutants to investigate plant-insect interactions (for example, Khan et al. [65] used *coi1* mutants and Ye et al. [26] used *aos* and *coi1* mutants), mutants were not utilized in this study to demonstrate the role of the JA pathway in plant-induced defense. Moreover, research in *Arabidopsis* has shown that priming the *WRKY29* transcription factor gene promoter through benzothiadiazole treatment is linked to H3K4me3, H3K4me2, H3K9ac, H4K5ac, H4K8ac, and H4K12ac; comparable results were obtained for *WRKY6* and *WRKY53* [80,83], implying that priming certain *WRKY* promoters involves histone modifications. These findings can guide future studies.

## Data Availability

Data are contained within the article and Supplementary Materials. The RNA-seq raw data has been submitted to the NCBI Short Read Archive (SRA) and has been assigned the accession number in the database.

## Supplementary Materials

The following supporting information can be downloaded at: https://www.mdpi.com/article/10.3390/plants13020273/s1, Figure S1: Effect of nitrogen supply levels on eggplant growth; Figure S2: Quality determination and differential gene analysis of RNA-Seq; Table S1: Real time PCR of differentially expressed genes from nitrogen sufficient and nitrogen deficient under WFT infestation for 24 h; Table S2: Primers of genes related to hormonal pathways are suitable for real-time qRT-PCR; Table S3: Hormone quantitative standard curve linear equation and correlation coefficient of detected substance by LC-MS/MS analysis; Table S4: Quantitative measurements of hormones associated with jasmonic acid (JA) and salicylic acid (SA) were taken in eggplant leaves inoculated with WFT at 0, 3, 9 and 24 h.
