# Supplementary material for: Nitrogen Deficiency Enhances Eggplant Defense against Western Flower Thrips via the Induction of the Jasmonate Pathway
Source: Plants (Basel). 2024 Jan 17;13(2):273. doi: 10.3390/plants13020273 (PMC10820576; doi:10.3390/plants13020273)
Supplement: Supplementary file 1 [file plants-13-00273-s001.zip › plants-2775024-supplementary.pdf]

Supplementary Material

Nitrogen Deficiency Enhances Eggplant Defense against Western Flower Thrips via the Induction of the Jasmonate Pathway

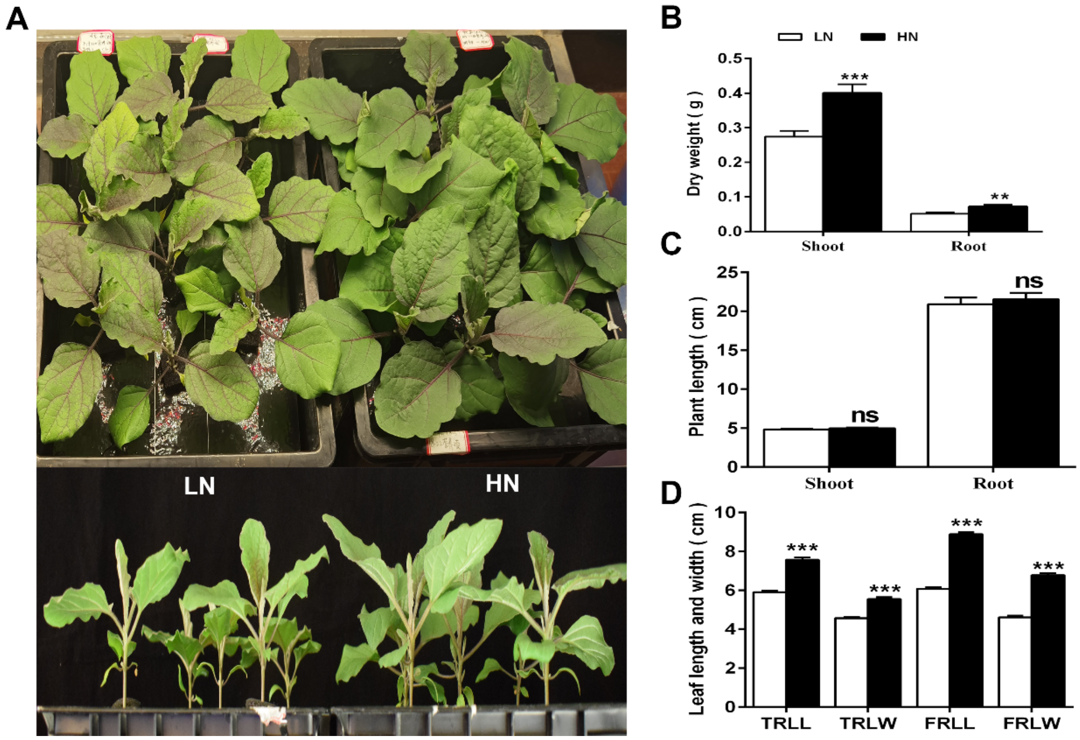

Supplementary Figure S1. Effect of nitrogen supply levels on eggplant growth. (A) Seedling morphology after nitrogen treatment. Fifteen-day-old seedlings were transplanted to nutrient solution containing 0.25mM $Ca(NO_3)_2$ (LN) or 2.5 mM $Ca(NO_3)_2$ (HN) and cultured for 5 days, and seedling morphology was taken. The dry weight (B), plant length (C) and leaf length and width (D) of eggplant shoot and root were determined. Mean ± SE based on 15, 15 and 20 biological duplications for dry weight, plant length and leaf length and width, respectively. Asterisks (*) indicate a significant difference from the indicated samples at *** $P < 0.01$ or *** $P < 0.001$ by Student's t-test*, ns means no significant difference. TRLL, the third real leaf length; TRLW, the third real leaf width; FRLL, the four real leaf length; FRLW, the four real leaf width.

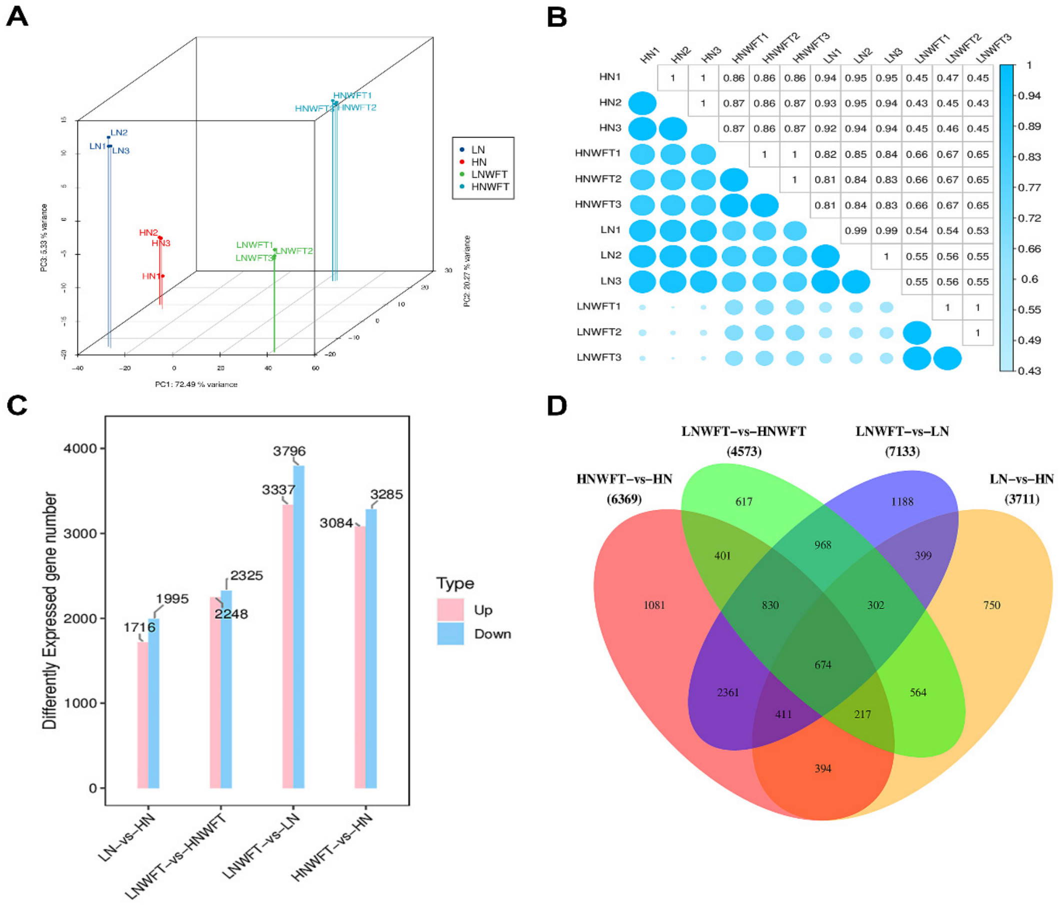

Supplementary Figure S2. Quality determination and differential gene analysis of RNA-Seq. The RNA-Seq metrics for quality control was shown as PCA analysis (A), correlation analysis (B) among LN, HN, LNWFT and HNWFT group. The overview of differential expressed gene under treatments was shown in (C). Venn diagram analysis of differential genes under treatment was shown in (D). LN, 0.25 mM $Ca(NO_3)_2$ cultured for 5 days; HN, 2.5 mM $Ca(NO_3)_2$ cultured for 5 days; LNWFT, 0.25 mM $Ca(NO_3)_2$ cultured for 5 days, then infested by *Frankliniella occidentalis* (WFT) for 24 h; HNWFT, 2.5 mM $Ca(NO_3)_2$ cultured for 5 days, then infested by WFT for 24 h.

Supplementary Table S1. Real time PCR of differentially expressed genes from nitrogen sufficient and nitrogen deficient under WFT infestation for 24 h.

		qPCR		RNA-Seq	
**Gene**	**Gene ID**	**Sequence (5′-3′)**	**FC**	**FC**	***p* value**
SmLOX1.4	smechr0800437	ggagggatcaaacttcctca<br>attccttcaccgtctgttcg	6.57	3.23	9.26E-11
SmACX1	smechr0802140	catttcacaacttggcactgga<br>ttagcacaggcaacagacatcc	4.33	2.90	8.77E-114
SmJAR1	smechr0400026	ccgagtcactgtcccatcca<br>gaagagttcaggaatcaagccata	10.60	4.41	8.00E-120
SmMYC1	smechr0800006	gggcgtatgctatcttctgg<br>tgttcttatcctcctcaccctta	2.64	2.24	6.09E-40
SmPAL1	smechr0500713	aatggcactgctgttggc<br>cttgatacatcaatcagagggttg	12.42	10.64	0
SmSPI5	smechr0302523	ctttatgtttacttccctttgtgg<br>aattgggttacctttcgtgtct	9.07	8.15	0
SmKTI5	smechr0502188	gtactacatacttcctgcccaca<br>cagattgaacaaccgatgagg	42.57	25.70	1.79E-61

Supplementary Table S2. Primers of genes related to hormonal pathways are suitable for real-time qRT-PCR.

Gene	Gene ID	Sequence (5′-3′)	Tm(℃)	Amplicon size(bp)
SmActin	GU984779.1	tggtcggaatgggacagaag	59.9	191
		ctcagtcaggagaacagggt	59.9	
SmLOX6	Smechr0300720	ggtattttgatgcctgtcgttat	56.6	154
		aaatggttgcgtcattagagc	56.1	
SmAOS3	Smechr0100287	gttcaatgcttatggtgggatg	58.2	96
		tcatttgccagccgagtgt	57.6	
SmAOC	Smechr0202404	atcaagcacgaagaggagaag	58.0	129
		ggtgacggcgaggtaagtg	61.9	
SmOPR3	Smechr0700225	gcctcggcttagcagttgtt	59.9	184
		gtcccttggtatgcgttcct	59.9	
SmEDS1	Smechr0602466	gcctaagcgttatcggttcac	60.0	198
		acatcattgccaaggaggttac	58.2	
SmSABP2	Smechr0300521	ggtaggacatagcatgggtggc	63.8	116
		caagattaaggtgaggagcaggc	62.0	
SmWRKY53	Smechr0802466	caaatgctcgcgaacttaca	55.8	168
		ggaataacccccaaccaaat	55.8	
SmMPK3	Smechr0600486	aactcccgcaacatccac	57.3	231
		tctgttcttctcctatcccttg	58.2	
SmMPK7	Smechr0202318	cttattgcagcgaatgctca	55.8	176
		gcatcatctcccgaatcact	57.8	

Supplementary Table S3. Hormone quantitative standard curve linear equation and correlation coefficient of detected substance by LC-MS/MS analysis.

Index	RT	Equation	r	Weighting
**JA**	5.77	y = 0.08013 x + 0.00434	0.99908	1 / x
**H_2JA**	6.14	y = 0.11283 x + 0.00364	0.99422	1 / x^2
**JA-Ile**	6.30	y = 0.30959 x + 0.00153	0.99886	1 / x^2
**JA-Phe**	6.36	y = 0.71968 x + 0.00763	0.99008	1 / x^2
**JA-Val**	5.98	y = 1.04346 x + 0.00282	0.99763	1 / x^2
**OPDA**	7.23	y = 1.88724 x + 0.05384	0.99026	1 / x^2
**MeJA**	6.88	y = 0.60087 x + 0.01723	0.99959	1 / x
**SA**	5.04	y = 0.10099 x + 0.01138	0.99994	1 / x
**SAG**	3.57	y = 0.03450 x + 0.00254	0.99503	1 / x^2

Supplementary Table S4. Quantitative measurements of hormones associated with jasmonic acid (JA) and salicylic acid (SA) were taken in eggplant leaves inoculated with WFT at 0, 3, 9 and 24 h.

Index	Group	N	Mean	SE	P
**H_2JA**	LN0	3	0.87932	0.066177	
**H_2JA**	HN0	3	0.59965	0.023926	0.016479*
**H_2JA**	LN3	3	0.91857	0.046153	
**H_2JA**	HN3	3	0.54944	0.008996	0.001423**
**H_2JA**	LN9	3	0.68557	0.028366	
**H_2JA**	HN9	3	0.61768	0.039876	0.237631ns
**H_2JA**	LN24	3	0.77136	0.018745	
**H_2JA**	HN24	3	0.55493	0.064682	0.032474*
**JA**	LN0	3	0.26558	0.026563	
**JA**	HN0	3	0.09045	0.0031134	0.002812**
**JA**	LN3	3	3.5483	0.16502	
**JA**	HN3	3	0.30993	0.03227	0.000043***
**JA**	LN9	3	10.863	0.42256	
**JA**	HN9	3	1.1597	0.13134	0.000026***
**JA**	LN24	3	41.112	1.861	
**JA**	HN24	3	0.36513	0.037979	0.000026***
**JA-Ile**	LN0	3	0.020587	0.002103	
**JA-Ile**	HN0	3	0.0074667	0.00087891	0.004517**
**JA-Ile**	LN3	3	2.9708	0.25201	
**JA-Ile**	HN3	3	0.15826	0.016319	0.007734**
**JA-Ile**	LN9	3	4.2715	0.23624	
**JA-Ile**	HN9	3	0.34255	0.02287	0.003348**
**JA-Ile**	LN24	3	9.6662	0.15061	
**JA-Ile**	HN24	3	0.16707	0.018999	0.000205***
**JA-Phe**	LN0	3	0.038957	0.0040445	
**JA-Phe**	HN0	3	0.01008	0.0011225	0.002339**
**JA-Phe**	LN3	3	0.22266	0.02573	
**JA-Phe**	HN3	3	0.068657	0.0083645	0.004705**
**JA-Phe**	LN9	3	0.082487	0.0019584	
**JA-Phe**	HN9	3	0.12046	0.013347	0.048077*
**JA-Phe**	LN24	3	0.21823	0.0029016	
**JA-Phe**	HN24	3	0.020547	0.0019797	5.9682E-7***
**JA-Val**	LN0	3	0	0	
**JA-Val**	HN0	3	0	0	0
**JA-Val**	LN3	3	0.063427	0.0075762	
**JA-Val**	HN3	3	0.0028267	0.00023362	0.015203*

Index	Group	N	Mean	SE	P
**JA-Val**	LN9	3	0.11148	0.011894	
**JA-Val**	HN9	3	0.00939	0.00078143	0.001021**
**JA-Val**	LN24	3	0.37536	0.016825	
**JA-Val**	HN24	0	0.0051467	0.00046484	0.000025***
**MeJA**	LN0	0	0	0	0
**MeJA**	HN0	0	0	0	
**MeJA**	LN3	0	0	0	0
**MeJA**	HN3	0	0	0	
**MeJA**	LN9	3	0.29339	0.027095	
**MeJA**	HN9	3	0	0	0.008422**
**MeJA**	LN24	0	4.45	0.18713	
**MeJA**	HN24	0	0	0	0.001764**
**OPDA**	LN0	3	0.71137	0.053394	
**OPDA**	HN0	3	0.12473	0.0084118	0.007061**
**OPDA**	LN3	3	1.4097	0.14901	
**OPDA**	HN3	3	0.12571	0.012138	0.001010**
**OPDA**	LN9	3	0.88578	0.11306	
**OPDA**	HN9	3	0.27133	0.012071	0.031014*
**OPDA**	LN24	3	2.1564	0.23671	
**OPDA**	HN24	3	0.23775	0.022316	0.001281**
**SA**	LN0	3	50.919	1.2887	
**SA**	HN0	3	43.618	1.0607	0.011926*
**SA**	LN3	3	133.16	4.5977	
**SA**	HN3	3	59.584	2.4878	0.000148***
**SA**	LN9	3	488.3	6.7789	
**SA**	HN9	3	137.07	2.5638	0.000001***
**SA**	LN24	3	450.86	10.023	
**SA**	HN24	3	126.25	6.4854	0.000011***
**SAG**	LN0	3	3456.5	213.67	
**SAG**	HN0	3	407.7	17.739	0.004645**
**SAG**	LN3	3	1983.3	211.93	
**SAG**	HN3	3	304.86	33.015	0.001440**
**SAG**	LN9	3	3805.1	237.02	
**SAG**	HN9	3	738.59	42.776	0.004785**
**SAG**	LN24	3	3178.4	47.493	
**SAG**	HN24	3	543.14	45.024	0.000002***

Asterisks (*) indicate a significant difference from the indicated samples at * $P < 0.05$, ** $P < 0.01$ or *** $p < 0.001$ by Student's t-test, ns means no significant difference.
